# Reduced Latency in the Metastatic Niche Contributes to the More Aggressive Phenotype of LM8 Compared to Dunn Osteosarcoma Cells

**DOI:** 10.1155/2013/404962

**Published:** 2013-12-04

**Authors:** Matthias J. E. Arlt, Ingo J. Banke, Josefine Bertz, Ram Mohan Ram Kumar, Roman Muff, Walter Born, Bruno Fuchs

**Affiliations:** Laboratory for Orthopedic Research, Department of Orthopedics, Balgrist University Hospital, University of Zurich, Forchstrasse 340, 8008 Zurich, Switzerland

## Abstract

Metastasis is the major cause of death of osteosarcoma patients and its diagnosis remains difficult. In preclinical studies, however, forced expression of reporter genes in osteosarcoma cells has remarkably improved the detection of micrometastases and, consequently, the quality of the studies. We recently showed that Dunn cells equipped with a *lacZ* reporter gene disseminated from subcutaneous primary tumors as frequently as their highly metastatic subline LM8, but only LM8 cells grew to macrometastases. In the present time-course study, tail-vein-injected Dunn and LM8 cells settled within 24 h at the same frequency in the lung, liver, and kidney of mice. Furthermore, Dunn cells also grew to macrometastases, but, compared to LM8, with a delay of two weeks in lung and one week in liver and kidney tissue, consistent with prolonged survival of the mice. Dunn- and LM8-cell-derived ovary and spine metastases occurred less frequently. *In vitro*, Dunn cells showed less invasiveness and stronger contact inhibition and intercellular adhesion than LM8 cells and several cancer- and dormancy-related genes were differentially expressed. In conclusion, Dunn cells, compared to LM8, have a similar capability but a longer latency to form macrometastases and provide an interesting new experimental system to study tumor cell dormancy.

## 1. Introduction

Tumor models in mice that make use of engrafted tumor cells are valuable tools for preclinical research in many types of cancer. Changes in tumor phenotypes in response to genetic manipulations in the engrafted tumor cells provide insight into the complex mechanisms of tumor pathogenesis and metastasis. Mouse tumor models also allow the preclinical testing of new drugs and treatment strategies designed to improve cancer therapy.

Mouse strain-specific syngeneic or xenotransplantation models in immunodeficient mice have also been established for osteosarcoma (OS), the most frequent primary bone tumor in children and young adults [1]. A frequently used syngeneic OS mouse model makes use of the murine Dunn OS cell line that has been isolated almost 50 years ago by Dunn and Andervont from a spontaneously developing OS primary tumor in a C3H mouse [2]. Subcutaneous (s.c.) reinjection of these cells into syngeneic C3H mice revealed fast growing primary tumors, but subsequent spontaneous metastasis to the lung, the predominant metastatic site in the human disease, was not observed. In the late 90s, Asai and Ueda succeeded in establishing a highly metastatic Dunn subline named LM8 [3]. It was selected *in vivo* according to the procedure of Poste and Fidler by serial reinjection of cells isolated from initially rare lung metastases [4]. LM8 cells, in contrast to the original Dunn cells, reproducibly disseminate from subcutaneous primary tumors to the lung and form multiple lung metastases with an incidence of 100% [3]. Thus, the LM8 model evolved to one of the most commonly used syngeneic OS mouse models for preclinical drug testing [1].

We recently reevaluated the *in vivo* metastatic properties of the original Dunn cells and compared it with those of the highly metastatic LM8 subline by making use of genetically manipulated cells that constitutively express the bacterial *lacZ* gene [5]. Tracking the metastasizing tumor cells in distant tissues and organs by X-gal staining down to the single cell level demonstrated for the first time that Dunn cells spontaneously metastasize from s.c. primary tumors to lungs and livers with the same incidence as LM8 cells. However, Dunn cells, different from LM8 cells, did not grow to macrometastatic foci and remained in the lung and the liver as micrometastases consisting of small cell clusters or single cells until the end of the study on day 25. These micrometastases remained undetectable with standard tissue staining techniques such as hematoxylin & eosin staining. These findings explain why the Dunn cells were so far considered as nonmetastatic.

Metastasis is a complex multistep process and multiple genes and factors regulate individual steps [6–8]. The different cellular processes along the metastatic cascade are also subject to variable regulation in time including periods of metastatic latency [9]. The results of the recent study that compared the metastatic potencies and properties of the LM8 and parental Dunn cells in C3H mice during an experimental period of 25 days raised the question whether the Dunn cells lacked cellular mechanisms needed for growth and development to macrometastases in the metastatic niche or whether they go through a longer period of metastatic latency than the LM8 cells upon arrival in the target organs.

Here, metastatic properties of Dunn and LM8 cells were further analyzed *in vitro*, and two follow-up studies were carried out *in vivo* to answer the question raised by the recent report [5]. In a first *in vivo* time-course study, *LacZ*-transduced Dunn and LM8 cells were intravenously (i.v.) injected into the tail vein of C3H mice and individual animals in both groups of mice were randomly selected and sacrificed at different time points up to 25 days after tumor cell injection. In a second survival study, C3H mice i.v. injected with *lacZ*-transduced Dunn and LM8 cells were examined until they became moribund and had to be sacrificed. In the time-course study, the lung, liver, and kidney and in the survival study, additionally, the urogenital tract and in some animals the spinal cords were analyzed for micro- and macrometastases by X-gal staining of respective organs and tissues upon sacrifice. Interestingly, Dunn cells formed macrometastases in the lung and in the liver and kidney with a delay of one and two weeks, respectively, compared to LM8 cells. The delay in the development of macrometastases was also reflected by significantly longer median survival of Dunn- compared to LM8-cell-injected mice. In conclusion, Dunn cells, much like LM8 cells, are equipped with all the cellular mechanisms needed for dissemination to different organs of C3H mice, but, different from LM8 cells, Dunn cells appear to go through a period of metastatic latency upon arrival in these organs.

## 2. Materials and Methods

### 2.1. Cell Lines

The mouse OS cell lines Dunn and LM8 were retrovirally transduced with a *lacZ* gene as described [5] and cultured at 37°C in DMEM/Ham F12 (1 : 1) medium (Invitrogen, Carlsbad, CA) supplemented with 10% heat-inactivated FCS in an incubator with a humidified atmosphere of 5% CO_2_.

### 2.2. RNA Extraction and Microarray Analysis

RNA isolation, preparation, and array hybridization were performed as described [10]. Briefly, total RNA isolated from Dunn and LM8 cells was quantified by measuring the absorption at 260 and 280 nm and RNA integrity was evaluated by agarose gel-electrophoresis. Complementary RNA preparation and array hybridization were performed by the Functional Genomics Center (Zurich, Switzerland) using Affymetrix Mouse Genome 430 2.0 (45101 probe sets) arrays. Raw data normalization and statistical analysis were performed using RACE (http://race.unil.ch/). The obtained data sets were then filtered by setting a fold-change cutoff >2 (both directions) and *P* < 0.05. Resulting data were then grouped into two sets, one containing the upregulated genes in LM8 compared to Dunn and the other containing the downregulated genes. Both sets of data were then analyzed for significant enrichment in certain Kyoto Encyclopedia of Genes and Genomes (KEGG) pathways via the Database for Annotation, Visualization and Integrated Discovery (DAVID) bioinformatics resources [11].

### 2.3. cDNA Synthesis and Real-Time PCR Analysis

1 *μ*g of total RNA was reverse-transcribed to cDNA using high capacity RNA-to-cDNA kit (Applied Biosystems, Foster City, CA, USA) according to the protocol provided by the manufacturer. Three independent RNA extracts from the individual cell lines were reverse-transcribed in a final volume of 10 *μ*L. Real-time PCR was carried out in StepOnePlus Real-Time PCR System (Applied Biosystems, USA) in 96-well plates. Primers for Mmp2 (FP: CGCTCAGATCCGTGGTGA; RP: CGCCAAATAAACCGGTCCTT), Bhlhb9 (FP: CCAGCCAGAGGGAAGAATAGC; RP: AAAGGCAGCAGAACACAAAGC), Fn1 (FP: CGAAGCCGGGAAGAGCAAG; RP: CGTTCCCACTGCTGATTTATCTG), Src (FP: TACCACTCCTCAGCCTGGAT; RP: ACACGAGGAAGGTGGATGTC), and Gapdh (FP: TGCAGTGGCAAAGTGGAGAT; RP: TTTGCCGTGAGTGGAGTCATA) were designed with the NCBI Primer BLAST software (http://www.ncbi.nlm.nih.gov/tools/primer-blast/). PCRs from individual RT reactions were carried out in triplicate. cDNA equivalent to 50 ng of RNA and appropriate primers were added to Power SYBR Green PCR Master Mix (Applied Biosystems, USA) and the samples were pre-incubated at 50°C for 2 min and at 95°C for 10 min and then subjected to 40 cycles of incubation at 95°C for 15 s and at 60°C for 1 min. The threshold for Ct values was set to 0.325. To verify the amplification of a single product in any of the PCR reactions, a melting curve was generated and analyzed after every run. Relative expression levels were calculated by the comparative cycle threshold (ΔΔCT) method and were normalized by GAPDH expression.

### 2.4. Western Blotting

Cells were grown to 80% confluence in normal medium. For supernatant (SN) preparation cells were starved over night with 2 mL of serum-free medium. The next day, the SN was collected, floating cells were removed by centrifugation, and the volume of the SN as well as the number of cells was determined. For western blotting the volume of the SN was adjusted to the total number of cells. SN samples were incubated with Laemmli sample buffer for 5 min at 95°C, then separated by SDS-PAGE, and subsequently transferred to nitrocellulose membranes. MMP2 was detected by incubation at 4°C overnight with a rabbit polyclonal anti-MMP2 primary antibody (Abcam) followed by incubation at RT for 1 h with secondary horseradish peroxidase conjugated goat anti-rabbit IgG (Santa Cruz Biotechnology, Inc.). The proteins were then visualized with Immobilon chemiluminescence substrate (Millipore) and quantified with a VersaDoc Imaging System (Bio-Rad Laboratories).

### 2.5. Assessment of Intercellular Adhesion and Contact Inhibition

For assessing intercellular adhesion the grade of cell clustering was determined in a first assay. Subconfluent Dunn and LM8 cells were detached with 1 mL Accutase (Sigma), counted in a hemocytometer, and adjusted to 250000 particles (>90% single cells) per mL. Two mL were then seeded into 6-well ultralow attachment plates (10 cm^2^; Costar) and four pictures per well of random fields were taken with a 4x objective (3.6 mm^2^) and a Zeiss Observer.Z1 microscope immediately after seeding and at different time points thereafter. Particle number analysis of pictures was performed using ImageJ. Clustering results in a decrease of particle number. After 25 h clusters were dissociated by pipetting 20 times up and down with a 1 mL Gilson pipet. The percent of clusters of >1000 pixels over total particles (>20 pixels) was then calculated. In a second assay intercellular adhesion was estimated by counting the number of single cells and of clusters of two or three cells in suspensions of trypsinized subconfluent and confluent cells. The percentage of single cells was calculated from the number of single cells and of cell clusters observed in three randomly selected fields of the hemocytometer. The total number of single cells or cell clusters counted in individual experiments varied between 100 and 150. To estimate the strength of intercellular adhesion, cells were seeded in 24-well plates at between 30% and 40% confluence and allowed to form a confluent monolayer. Six wounds per cell line and experiment were then generated with a sterile pen with a tip diameter below 1 mm. The wound widths were then measured using a Nikon Diaphot TMD microscope with a 10x objective and a 10x ocular with a graded 1 mm scale. Contact inhibition was assessed by counting the total number of cells grown to subconfluence or visual confluence in 25 cm^2^ tissue culture flasks. The cells were trypsinized and aliquots of cell suspensions were counted in a hemocytometer.

### 2.6. Three-Dimensional Matrigel Degradation and Migration Assay

Subconfluent cells were trypsinized and resuspended in ice-cold cell culture medium without FCS. The cell density was estimated with a hamocytometer and adjusted to 500 cells per *μ*L. Equal volumes of ice-cold cell suspension and Matrigel (10 mg/mL) were mixed and 25 *μ*L were applied to the center of a 6-well plate in triplicate. After gelling at 37°C for 30 min, 2 mL of cell culture medium supplemented with 10% FCS was added. Evasion foci outside the the Matrigel boarders, which resulted from combined migration and degradation of Matrigel matrix, were counted under a microscope. Evasion events per drop were proportional to the number of cells per drop, and evasion occurred earlier at higher cell densities of seeded cells (not shown).

### 2.7. Time Course of Dunn and LM8 Experimental Metastasis

Eight-week-old female C3H/HeNCrl (C3H) mice were obtained from Charles River Laboratories (Sulzfeld, Germany) at least 10 days before the beginning of the experiment. Housing conditions and experimental protocols were in accordance with the guidelines of the “Schweizer Bundesamt für Veterinärwesen” and approved by the authorities of the Canton of Zurich. On day 0 of the experiment, 10^6^ *lacZ*-transduced Dunn or LM8 cells in 200 *μ*L PBS were injected into the tail vein of the mice. The health of the mice was monitored daily. On days 1, 4, 7, 14, 21, and 25 after tumor cell injection 4 randomly selected mice per group were sacrificed and selected tissues and organs were analyzed for the presence of metastases as recently described [5]. Briefly, blood was removed from the lungs by perfusion with PBS under anesthesia with ketamine, xylazine, and acepromazine. The lungs were then fixed *in situ* for 10 min under inflation with 3% paraformaldehyde (PFA). Subsequently, the lungs, livers, and kidneys were removed, fixed with 2% PFA and 0.2% glutaraldehyde in PBS at room temperature for 30 min, and then washed with PBS and stained with X-gal solution (Enzo Life Sciences AG, Lausen, Switzerland) at 37°C for at least 3 hours. Photographs of whole lungs and livers were taken with an E620 DSLR camera under an SZX 10 binocular microscope (Olympus Corporation, Tokyo, Japan) and imported as JPEG files into PowerPoint software. Close-ups of organ surfaces were taken with a Kappa PS 20 C digital camera (Kappa opto-electronics GmbH, Gleichen, Germany) attached to an Eclipse E600 microscope (Nikon Corporation, Tokyo, Japan) and imported as TIFF files into PowerPoint software. Due to the great convexity and intense colouring of the kidney, close-ups of micrometastases on its surface could not be taken.

### 2.8. Survival Experiment with the Dunn and LM8 Experimental Metastasis Models

The mouse strain and source, the housing conditions, the number of tumor cells, and the route of tumor cell injection were those described above. In this experiment, the metastases-bearing mice (8 per group) were kept alive as long as possible and were sacrificed individually when they became moribund. The lung, liver, and kidneys as well as other selected organs (ovaries, spine) were then also prepared and analyzed as described.

### 2.9. Statistical Analysis

The *in vitro* data were statistically analyzed with the two-tailed paired student's *t*-test and the Kaplan-Meier survival curves were statistically analyzed with the log rank (Mantel-Cox) test and the Gehan-Breslow-Wilcoxon test. Data were considered significantly different when *P* < 0.05.

## 3. Results

### 3.1. Differences between Dunn and LM8 Cells in Intercellular Adhesion, Contact Inhibition, and Invasiveness *In Vitro*

**Figure 1:**
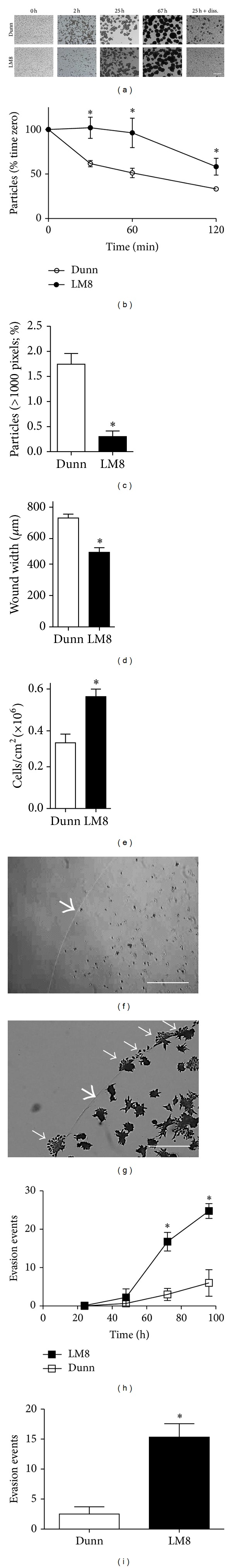
Intercellular adhesion, contact inhibition, and invasiveness of Dunn and LM8 cells *in vitro*. (a) Representative time course of intercellular adhesion and dissociation of Dunn (upper panels) and LM8 (lower panels). Scale bar: 400 *μ*m. (b) Quantification of particle number in percent of the 0 min value. Particle numbers at time zero were 1036 ± 105 and 1313 ± 93 (*P* > 0.05) in Dunn and LM8, respectively, and were set to 100%. (c) Dissociation by pipetting after 25 h association. Results are the means ± SEM of 3 independent experiments. (d) Intercellular adhesion was further estimated by wounding of confluent cells grown in 24-well plates with a pen and subsequent measurement of the width of the fresh wound as described in Section 2. (e) The cells were grown in 25 cm^2^ flasks to visual confluence and then trypsinized and counted to estimate differences in contact inhibition. The invasive activity of cells *in vitro* was determined in a three-dimensional Matrigel degradation and migration assay. Representative microscopic images of LM8 cells seeded in Matrigel drops (500 cells/*μ*L) immediately after gelling (f) and after incubation for 72 h in cell culture medium (g) are shown (scale bars in (f) and (g): 500 *μ*m). Bold arrows in (f) and (g) point to the border of the Matrigel drop, and normal arrows in (g) to foci evading from the Matrigel drop from cell clusters growing inside the Matrigel drop. (h) The number of foci evading from the Matrigel drops (*n* = 3) was counted daily. (i) The difference between the numbers of Matrigel-evading Dunn and LM8 cell foci per drop (*n* = 4) peaked at 72 h after cell seeding. All results are shown as the means ± SEM of 3–6 independent experiments. Asterisks (∗) indicate statistically significant difference (*P* < 0.05).

Loss of intercellular adhesion and contact inhibition as well as the ability to degrade and migrate through extracellular matrix are prerequisites for metastasizing tumor cells to colonize distant organs. In the present study, we found that after seeding the same number of >90% solitary cells in ultralow attachment plates the Dunn cells (upper panel in Figure 1(a)) are aggregated much faster than the LM8 cells (lower panel in Figure 1(a)) to clusters resulting in a significantly higher (*P* < 0.05) aggregation rate within the first two hours (Figure 1(b)). After 1 day both cell lines had formed rounded clusters with mean areas of 3509 ± 547 *μ*m^2^ for Dunn and 3188 ± 373 *μ*m^2^ for LM8 which further increased significantly until day 3 to 6065 ± 800 *μ*m^2^ (*P* < 0.002) and 5241 ± 576 *μ*m^2^ (*P* < 0.04). Interestingly, after 1 and 3 days the clusters formed by Dunn cells were more regular (round spheres) than those formed by the LM8 cells (Figure 1(a)). Dissociation of clusters after one day (last picture in both panels of Figure 1(a)) revealed a lower amount (*P* < 0.004) of big clusters in LM8 compared to Dunn (Figure 1(c)). Furthermore, when the percentage of single cells was assessed in trypsinized populations derived from confluent Dunn and LM8 cells, it was found to be 17% higher (*n* = 5; *P* < 0.003) in LM8 than in Dunn cultures (data not shown). These observations suggested that, in confluent cultures, intercellular adhesion between Dunn cells was stronger than that between LM8 cells. These observations were also in agreement with those made in a wounding assay. There, the widths of wounds applied to LM8 cell monolayers were 32% (*n* = 6; *P* < 0.0002) smaller than those found in wounds of Dunn cell monolayers (Figure 1(d)), indicating again a significantly decreased strength in intercellular adhesion between LM8 compared to Dunn cells. In addition, the cell density of LM8 cells at visual confluence was 70% higher than that of Dunn cells (*n* = 5; *P* < 0.005) implying a significant loss of contact inhibition of LM8 compared to Dunn cells (Figure 1(e)).

The ability of Dunn and LM8 cells to degrade and to migrate in a three-dimensional matrix was evaluated with a Matrigel drop assay. Immediately and up to 24 h after cell seeding cells that had migrated out of the Matrigel drops were not observed (Figure 1(f)). At later time points, evasion foci were visible on the surface of the Matrigel drops, indicating that the OS cells had locally degraded Matrigel and had migrated to and slightly beyond the surface of the drop (Figure 1(g)). The number of evasion foci continuously increased up to 96 h (Figure 1(h)). After longer incubation periods, adjacent foci began to fuse and were no longer quantifiable. Two days after cell seeding the number of LM8 evasion foci increased much faster than that of Dunn cells and the largest difference was observed at 72 h when LM8 cells had formed a 6 times higher number of evasion foci than Dunn cells (*P* < 0.003; Figure 1(i)). The results indicate a higher mobility and matrix degrading capacity of LM8 compared to Dunn cells.

### 3.2. Gene Expression Analysis of Dunn and LM8 Cell Lines Reveals Differentially Regulated Genes in “Pathways in Cancer” (KEGG Pathway) and among Dormancy Genes

There is good evidence that disseminated tumor cells, which have acquired all necessary properties to mature to macrometastatic foci in the metastatic niche of distant organs, may preserve those properties when they are isolated and cultured *in vitro* [9, 12]. Based on this assumption, we performed microarray analysis of the Dunn and LM8 cell lines and subsequently analyzed the differences in their gene expression profiles. In total, 1257 gene sets were differentially regulated (>2-fold change; *P* < 0.05) in Dunn and LM8 cells. The DAVID program detected enrichment of these regulated gene sets in 17 KEGG pathways (Table 1). The highest gene enrichment was found for “Pathways in Cancer” (3.78%) followed by “Focal Adhesion” (2.68%), “Cytokine-Cytokine Receptor Interaction” (2.31%), and “Regulation of Actin Cytoskeleton” (1.95%). From the 31 gene sets that were associated with cancer pathways, 19 were significantly down- and 12 significantly upregulated in the highly metastatic LM8 cells compared to the low-metastatic Dunn cells (Table 2). Interestingly, the downregulated genes were not only predominant in number but also with regard to fold change. While the upregulation in LM8 cells was highest for matrix metallopeptidase 2 (Mmp2) gene transcripts (~7-fold), the most pronounced downregulation (~24-fold) was found for kit ligand (Kitl) gene transcripts. The fold change in the top 5 of the downregulated genes (Kitl, Pparg, Vegfc, Ptgs2, and Pdgfb) ranged from 24.57 to 5.95 and in the top 5 of the upregulated genes (Mmp2, Dapk2, Tcf7, Wnt1, and Birc3) from 6.91 to 3.18.

In a second analysis we checked our microarray data for differential regulation of genes that are likely involved in tumor cell dormancy [13–15]. For 7 out of 44 investigated genes (described in [13–15]) the expression was significantly different between Dunn and LM8 cells (>2-fold change; *P* < 0.05; Table 3). Among those genes, the connective tissue growth factor (Ctgf) gene was the only one found upregulated (2.72-fold) in LM8 compared to Dunn cells. Basic helix-loop-helix domain containing class B9 (Bhlhb9) with a 61-fold decrease was one of the most heavily downregulated genes in LM8 compared to Dunn cells. The fibronectin 1 (Fn1), Rous sarcoma oncogene (Src), transforming growth factor beta 2 (Tgfb2), transforming growth factor beta receptor 1 (Tgfbr1), and tropomyosin 1 alpha (Tpm1) genes, on the other hand, were downregulated only 2-3-fold in LM8 compared to Dunn cells.

**Figure 2:**
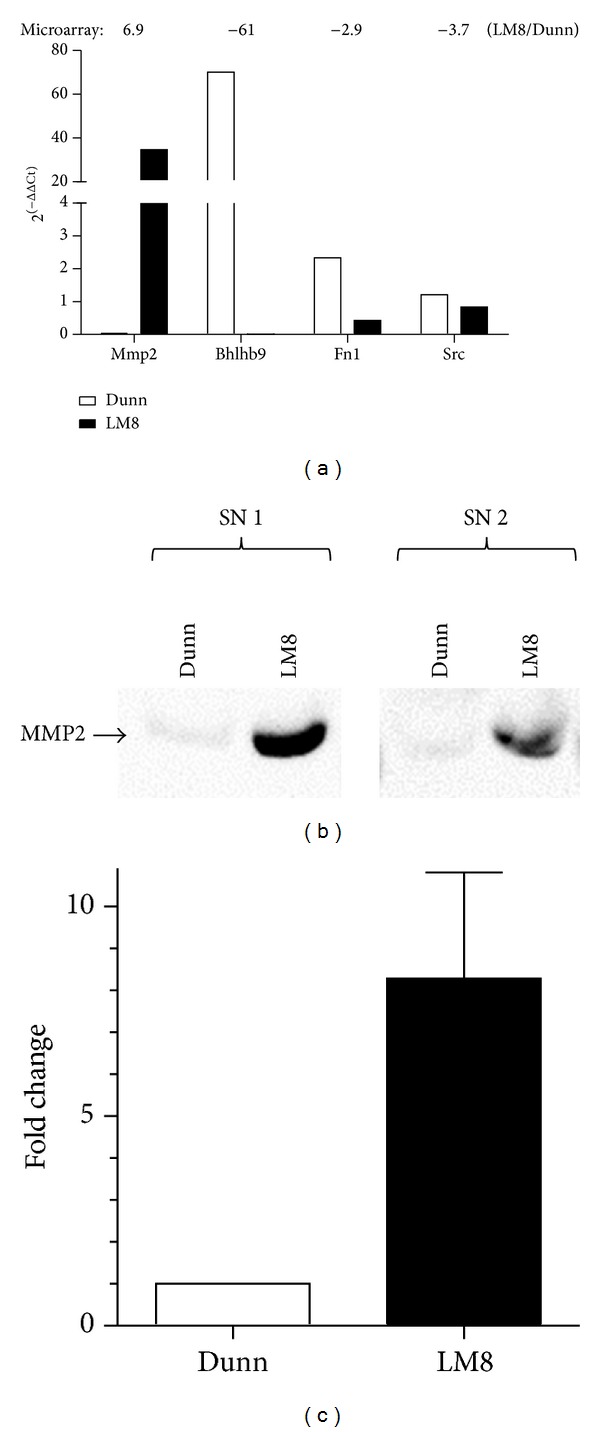
(a) qRT-PCR analysis of Mmp2, Bhlhb9, Fn1, and Src mRNA expression normalized to Gapdh in Dunn and LM8 cells. For comparison the fold-change values calculated from the microarray data are indicated above the graph. (b) Representative western blots of MMP2 protein levels in FCS-free supernatant of Dunn and LM8 cells and (c) quantitative analysis of three independent western blots experiments. Results are shown as the means ± SEM.

For selected genes (Mmp2, Bhlhb9, Fn1, and Src) the data of the microarray were confirmed by qRT-PCR (Figure 2(a)). For Mmp2, Bhlhb9, and Fn1 the difference in expression of the individual genes between Dunn and LM8 cells was even more pronounced than in the microarray analysis. Furthermore the protein expression of MMP2 was analyzed in conditioned medium. The western blot analysis revealed 8-fold higher MMP2 levels in FCS-free supernatant of LM8 cells than in supernatant of Dunn cells (Figures 2(b) and 2(c)).

### 3.3. Delayed Formation of Macrometastases by Dunn Compared to LM8 Cells in C3H Mice Is Associated with Prolonged Survival

**Figure 3:**
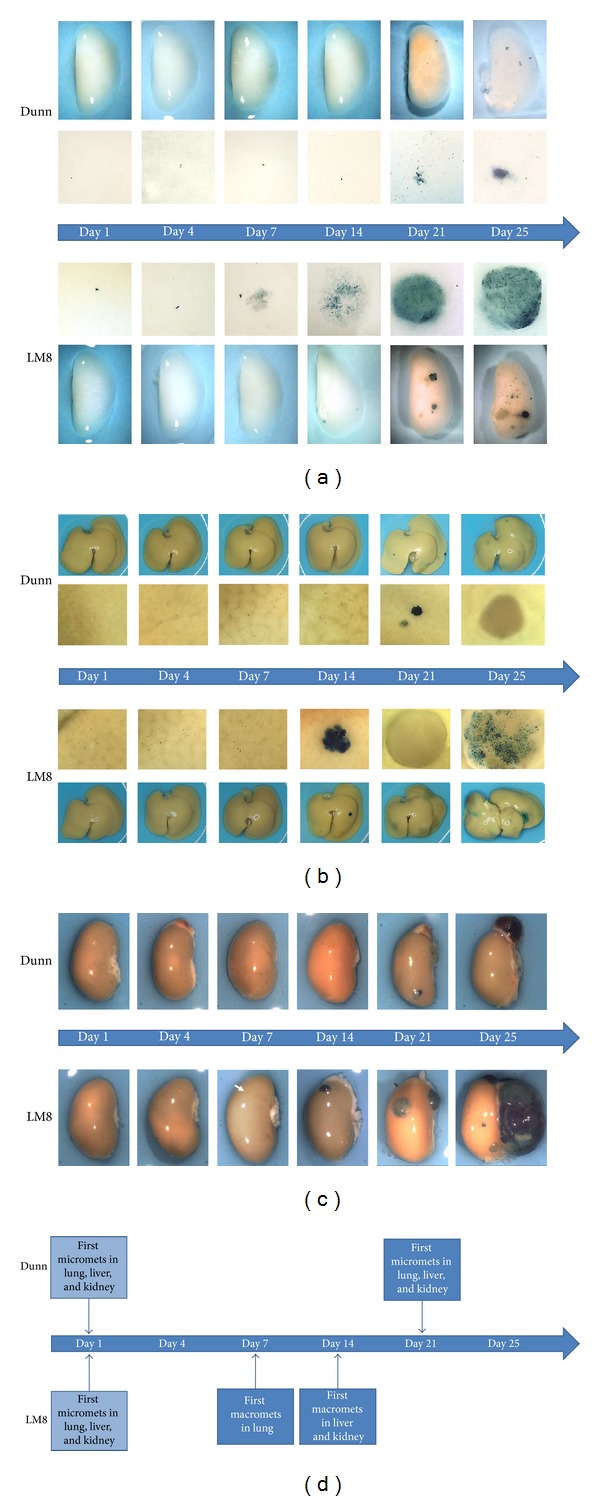
Appearance over time of experimental metastases in indicated organs after intravenous injection of *lacZ*-transduced Dunn and LM8 cells in C3H mice. ((a)–(c)) Images show X-gal-stained metastases in blue in representative whole mounts and ((a), (b)) corresponding close-ups of lungs (a), livers (b), and kidneys (c) collected from mice that were sacrificed at indicated time points after tumor cell injection. (d) Schematic summary of the first appearance of micro- and macrometastases (>0.1 mm) over time in indicated organs.

Potential differences in the outgrowth of Dunn- and LM8-cell-derived lung metastases were investigated in a time-course study that monitored the formation of micro- and macrometastases over time in selected organs of C3H mice upon i.v. injection of 1 × 10^6^ *LacZ*-transduced Dunn or LM8 cells. Micrometastases of both cell lines, consisting of single cells or cell clusters smaller than 0.1 mm in diameter, were already observed one day after i.v. tumor cell injection (TCI) in the lungs (Figure 3(a), first set of close-up images in the upper and lower panels) and on the liver (Figure 3(b)) and kidney (Figure 3(c)) surfaces. On day 4 after tumor cell injection the metastatic pattern in the organs of both, Dunn and LM8-cell-injected mice, appeared unchanged. However, one week after TCI the first metastatic foci larger than 0.1 mm, considered as macrometastases, became visible in the lungs of mice injected with LM8 cells (Figure 3(a), lower panel). Macrometastases in the livers and kidneys of LM8-injected mice were first detected in animals that were sacrificed two weeks after TCI (lower panels in Figures 3(b) and 3(c)). In mice injected with Dunn cells, on the other hand, analyzed organs still contained only micrometastases at the end of weeks one and two of the experiment (upper panels in Figures 3(a)–3(c)). But, interestingly, three weeks after Dunn cell injection, the lungs (Figure 3(a), upper panel), livers, and kidneys (upper panels in Figures 3(b) and 3(c)) of the mice showed for the first time outgrowing macrometastases. However, these Dunn-cell-derived macrometastases were considerably smaller than those observed at the same time in the lungs, livers, and kidneys of LM8-injected mice. Apparently comparable continuous growth of respective macrometastases maintained the observed differences in size until the end of the study on day 25. Interestingly, in both, the Dunn and the LM8-cell-injected C3H mice, the final size of respective liver and kidney macrometastases was larger than that of the respective lung macrometastases. In LM8-injected mice this was an unexpected observation because, as noted before, macrometastases in the livers and kidneys developed with one week delay compared to those in the lungs.  Figure 3(d) schematically summarizes the results obtained in this time-course study.

**Figure 4:**
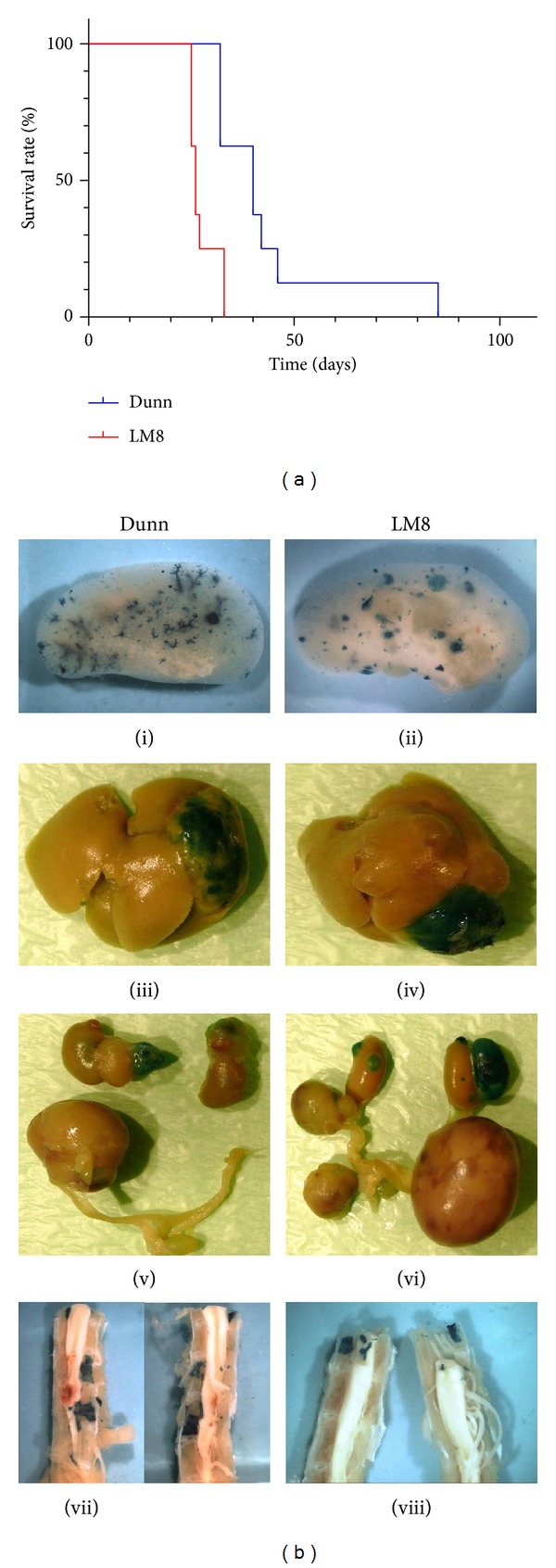
Animal survival and tissue distribution of metastases at sacrifice in mice intravenously injected with *lacZ*-transduced Dunn or LM8 OS cells. (a) Kaplan-Meier survival curves of C3H mice injected with LM8 (red line) or Dunn (blue line) cells. (b) Metastatic pattern in lung (i-ii), liver (iii-iv), urogenital tract (v-vi), and spine (vii-viii) of moribund mice at sacrifice.

The additional survival study was performed to investigate the effect of the delayed outgrowth of Dunn-compared to LM8-cell-derived macrometastases on the survival of the respective mice. C3H mice were i.v. injected with the same number of tumor cells as in the time-course study and sacrificed when they became moribund.  Figure 4(a) shows the corresponding Kaplan-Meier curves. In the group of mice injected with LM8 cells, the first 3 animals had to be sacrificed on day 25 after TCI, and the median survival time in this group was 26 days. The last two mice of this group became moribund on day 33. The first mice in the group of animals injected with Dunn cells, on the other hand, had to be killed on day 32 and the median survival time was 40 days. The last mouse in this group was sacrificed on day 85. Overall, these results revealed a statistically significant difference (*P* < 0.005) in the survival of the two groups of mice. Analysis of the inner organs of moribund Dunn- and LM8-injected mice confirmed the faster growth to macrometastases of the two cell lines in the liver (Figure 4(b) (iii-iv)) and kidney (Figure 4(b) (v-vi)) than in the lung (Figure 4(b) (i-ii)). In addition, massive metastases were also found in several ovaries of Dunn-*LacZ*- (Figure 4(b) (v)) and LM8-*LacZ*-injected mice (Figure 4(b) (vi)). Furthermore, a few mice of both groups showed paralysis of the hindlimbs. Postmortem analysis of their spinal columns revealed metastases in the vertebral bodies (Figure 4(b) (vii-viii)). Some of these metastases were so large that they had already infiltrated the lumen of the spine leading to compression of the spinal cord, which was most likely the cause of the paralysis.

## 4. Discussion

Metastases are the major cause of death in OS. A better understanding of the cellular mechanisms involved in the multistep metastatic processes and early diagnosis of metastatic disease are therefore of importance for an adequate therapy on time and might result in improved survival of the patients. Unfortunately, although all disseminating tumor types employ largely similar mechanisms in the multistage process of local tissue invasion, intravasation, survival in the circulation, extravasation, and colonization of distant organs, their disseminating activities differ considerably in frequency, direction, and temporal course. Even within the same type of cancer these characteristics can differ depending on the genetic changes acquired by the tumor cells during the metastatic process [9, 12].

Recently, we have been able to demonstrate that, different from what was initially reported, s.c. injected mouse Dunn OS cells disseminate to the lung and liver with the same frequency as their highly metastatic derivatives LM8, but, different from LM8 cells, Dunn cells did not further develop to macrometastases during the experimental period of 25 days [5]. Based on this novel so far not reported disseminating phenotype of the Dunn OS cells, we performed in the present study experiments *in vitro* and *in vivo* to further characterize the metastatic capabilities of Dunn compared to LM8 cells. Metastatic properties such as contact inhibition, intercellular adhesion, and invasiveness were examined *in vitro*. *In vivo* time-course and survival studies were designed to answer the question whether Dunn cells that infiltrate lung or liver tissue will never grow to macrometastases or even disappear or only remain latent for a certain time as single cells or small cell clusters before they restart excessive proliferation and form overt metastases as it has been described for, for example, breast cancer cells [16, 17].

The results of the *in vitro* studies revealed indeed a putative milder metastatic phenotype for the Dunn cells, for example, less invasiveness and stronger contact inhibition and intercellular adhesion, than observed for the LM8 cells. Interestingly, the here observed significantly higher aggregation rate and faster formation of spheres of Dunn compared to LM8 cells in ultralow attachment plates are in good accordance with experiments in soft agar assays in the previous study which indicated a faster anchorage independent growth of Dunn compared to LM8 cells [5]. These findings taken together suggest that factors other than proliferation rates might determine the metastatic growth of the two cell lines. This is in agreement with another study where LM8 cells overexpressing different cadherins showed in comparison to the wild type LM8 massively reduced formation of lung metastases *in vivo* but similar proliferation rates *in vitro* [18]. The differences in invasiveness between the Dunn and LM8 cells observed in the present study in the Matrigel drop assay are in agreement with the findings of Asai et al. who demonstrated higher MMP-2 expression and activity in LM8 than in Dunn cells [3], which we were able to confirm in the present study with our microarray, qRT-PCR, and western blot data. It is conceivable that the different capabilities of Dunn and LM8 cells to degrade and penetrate extracellular matrix might be more important for infiltration and colonization of target organs than for the escape from the primary tumor. At the primary tumor site, cancer cells are usually quite well supported by recruited stromal and immune cells, which substantially contribute to, for example, local degradation of the ECM by expressing several proteases [19, 20]. In distant organs they might be much more dependent on their own capabilities to invade healthy tissue. However, the ability of disseminating tumor cells to invade the tissue of distant organs is only a prerequisite but not sufficient for the formation of overt metastases. It is known that even certain premalignant cell lineages have the capacity to disseminate, infiltrate distant organs, and survive in tissues different from their origin [21]. Tumor cells therefore have to acquire additional properties to achieve full metastatic competence [9]. Our microarray data indicated that under *in vitro* culture conditions 31 genes associated with cancer pathways and 7 genes involved in tumor cell dormancy were differentially regulated in the low-metastatic Dunn compared to the highly metastatic LM8 cells. Interestingly, almost 2/3 of the genes involved in cancer pathways and 6 out of 7 dormancy-related genes were expressed at lower levels in the highly metastatic LM8 than in the low-metastatic Dunn cells, suggesting that the downregulation of certain genes is of equal or even higher importance than the upregulation of others. This is further supported by the observation that the fold decrease of many downregulated genes was much higher than the fold increase of most upregulated genes. Bhlhb9 was among the top 7 most heavily regulated genes recognized in this microarray analysis. Its product belongs to the basic helix-loop family of transcription factors, which is involved in tumorigenesis-associated gene regulation [22]. Members of this family, for example, BHLHB3, induce growth arrest of disseminated tumor cells without suppressing primary tumor growth [23] and BHLHB9 itself might play a pivotal role in apoptotic cell death [24]. PPARG, also strongly downregulated in LM8 cells, belongs to the nuclear hormone receptor superfamily and, as a tumor repressor gene, it is silenced or mutated during tumorigenesis of, for example, sporadic colorectal cancer and its down-regulation/inactivation correlates significantly with the aggressiveness of the disease [25]. Interestingly, the expression of quite a few angiogenesis- and metastasis-related genes, particularly growth factors, was also found downregulated in LM8 compared to Dunn cells. It is known that many cancer-associated gene products have bifunctional roles in tumor progression and metastasis, for example, TGF**β**2 and TGF**β**R1. TGF**β** is often prominently expressed in the tumor microenvironment and promotes beside tumor growth also metastasis by inducing the expression of genes important for the dissemination of tumor cells [26]. In distant organs, on the other hand, its autocrine/paracrine signaling might by considerably involved in induction and maintenance of dormancy in disseminated tumor cells [14].

The results of the *in vivo* experimental metastasis studies are in good agreement with these microarray results and the findings obtained *in vitro* since they showed that the Dunn cells, much like the LM8 cells, are capable of growing to macrometastases in the lung, the liver, and the kidney and in some mice even in ovaries and vertebral bodies of the spine, but with a considerable delay when compared to LM8 cells. Thus, the Dunn OS cells are not only able to disseminate from primary tumors to distant organs as reported in the previous study [5] but also grow to macrometastases, but during a phase of dormancy they need to acquire some additional properties in the new tissue microenvironments. In this context it is also important to note that in LM8-cell-injected mice lung macrometastases occurred one week earlier than those recognized in the livers and kidneys, despite the fact that all these organs contained micrometastases as early as one day after i.v. injection of LM8 cells. This indicates that the LM8 cells that were initially selected for a high lung metastatic potential apparently needed some extra time to adapt to the liver or kidney microenvironments before they started to grow to macrometastases in these organs. They may have acquired a lung-specific tropism during their generation by several cycles of *in vivo* selection of Dunn-cell-derived lung metastases. Such organ-specific metastatic phenotypes have been reported and can also be stably maintained *ex vivo* [12]. However, detailed molecular mechanisms that provide a lung-specific tropism remain to be investigated. Surprisingly, at the end of the study, liver and kidney macrometastases in both the Dunn and the LM8-cell-injected mice were by far larger in size than the respective lung metastases. These liver and kidney macrometastases, besides occasional ovary and spine metastases, were likely also the life-threatening metastases for the mice investigated in the survival study.

Altogether, the results of the *in vivo* studies unequivocally showed that the Dunn OS cells are also capable of growing to macrometastases in different tissue environments, but with some latency when compared to LM8 cells, which resulted in prolonged survival. The results further indicate that, after acquisition of a potentially organ-specific colonization competence, both the Dunn and LM8 OS cells proliferated unrestrictedly in the different organs.

In conclusion, the results of the present study provide evidence for faster adaptation of the highly metastatic mouse LM8 OS cells than of the parental Dunn OS cells to the requirements for colonization of distant tissues after dissemination from the primary tumors. Apart from that, Dunn and LM8 OS cells exhibit a comparable metastatic phenotype in syngeneic C3H mice. Consequently, the Dunn OS cell line can no longer be considered as non- or low metastatic and it provides an interesting new experimental system to study tumor dormancy.

## Figures and Tables

**Table 1:** Number of regulated (>2-fold; *P* < 0.05) Dunn/LM8 gene sets enriched in KEGG pathways (DAVID).

Pathways	Gene count	%^1^
Pathways in cancer	31	3.78
Focal adhesion	22	2.68
Cytokine-cytokine receptor interaction	19	2.31
Regulation of actin cytoskeleton	16	1.95
Axon guidance	14	1.71
Cell adhesion molecules (CAMs)	14	1.71
Vascular smooth muscle contraction	11	1.34
ECM-receptor interaction	10	1.22
Apoptosis	9	1.10
Basal cell carcinoma	8	0.97
Complement and coagulation cascades	8	0.97
Adherens junction	8	0.97
Hematopoietic cell lineage	8	0.97
Hedgehog signaling pathway	7	0.85
Glutathione metabolism	6	0.73
Prion diseases	5	0.61
Glycosphingolipid biosynthesis	4	0.49

^1^Proportion of all regulated gene sets.

**Table 2:** Genes involved in cancer pathways (KEGG pathway; DAVID) and whose expression is significantly down- or upregulated in LM8 compared to Dunn cells.

	Symbol	Fold change	*P* value
Genes downregulated in LM8 cells			
Kit ligand	Kitl	−24.57	4.26*E* − 12
Peroxisome proliferator activated receptor gamma	Pparg	−15.70	2.02*E* − 10
Vascular endothelial growth factor C	Vegfc	−8.20	3.50*E* − 09
Prostaglandin-endoperoxide synthase 2	Ptgs2	−7.46	4.34*E* − 08
Similar to platelet-derived growth factor B chain; platelet-derived growth factor, B polypeptide	Pdgfb	−5.95	1.36*E* − 09
Insulin-like growth factor 1	Igf1	−4.70	3.88*E* − 08
Aryl hydrocarbon receptor nuclear translocator 2	Arnt2	−3.80	1.16*E* − 05
Endothelial PAS domain protein 1; similar to endothelial PAS domain protein 1	Epas1	−3.62	1.13*E* − 05
Fibronectin 1	Fn1	−2.95	5.69*E* − 08
Integrin alpha 3	Itga3	−2.79	1.02*E* − 07
Integrin alpha 6	Itga6	−2.78	1.89*E* − 07
Laminin B1 subunit 1	Lamb1-1	−2.64	8.26*E* − 05
Transforming growth factor, beta 2	Tgfb2	−2.43	6.09*E* − 06
RAS-related C3 botulinum substrate 3	Rac3	−2.37	1.65*E* − 04
Bone morphogenetic protein 4	Bmp4	−2.26	8.64*E* − 07
Signal transducer and activator of transcription 3; similar to Stat3B	Stat3	−2.23	5.64*E* − 06
Transforming growth factor, beta receptor I	Tgfbr1	−2.15	5.06*E* − 06
Fibroblast growth factor receptor 2	Fgfr2	−2.14	9.69*E* − 07
Interleukin 6	Il6	−2.01	5.78*E* − 05
Genes upregulated in LM8 cells			
Matrix metallopeptidase 2	Mmp2	6.91	4.24*E* − 09
Death-associated protein kinase 2	Dapk2	4.70	7.79*E* − 10
Transcription factor 7, T-cell specific	Tcf7	3.82	1.21*E* − 06
Wingless-related MMTV integration site 1	Wnt1	3.24	2.55*E* − 07
Baculoviral IAP repeat-containing 3	Birc3	3.18	2.57*E* − 08
Patched homolog 2	Ptch2	3.02	1.66*E* − 05
Frizzled homolog 7 (*Drosophila*)	Fzd7	2.94	7.75*E* − 07
Wingless-related MMTV integration site 10a	Wnt10a	2.64	1.93*E* − 05
Fibroblast growth factor 15	Fgf15	2.63	2.79*E* − 05
GLI-Kruppel family member GLI2	Gli2	2.59	1.62*E* − 05
Similar to wingless-related MMTV integration site 8b; wingless-related MMTV integration site 8b	Wnt8b	2.39	3.89*E* − 04
Predicted gene 10124; predicted gene 6340; CDC28 protein kinase 1b	Cks1b	2.29	1.24*E* − 06

Genes with >2 times decrease or increase in expression and a *P* value < 0.05 were selected and applied to the DAVID program.

**Table 3:** Regulation of potential dormancy genes in LM8 compared to Dunn cells.

	Symbol	Fold change	*P* value
Genes downregulated in LM8			
Basic helix-loop-helix domain containing, class B9	Bhlhb9	−60.98	8.46*E* − 13
Fibronectin 1	Fn1	−2.95	5.69*E* − 08
Transforming growth factor, beta 2	Tgfb2	−2.43	6.09*E* − 06
Rous sarcoma oncogene	Src	−2.36	2.24*E* − 06
Tropomyosin 1, alpha	Tpm1	−2.28	2.53*E* − 07
Transforming growth factor, beta receptor I	Tgfbr1	−2.15	5.06*E* − 06
Genes upregulated in LM8			
Connective tissue growth factor	Ctgf	2.72	2.18*E* − 04
Genes not differentially regulated in LM8			
Secreted phosphoprotein 1	Spp1	−1.78	2.08*E* − 05
A kinase (PRKA) anchor protein (gravin) 12; SSeCKS	Akap12	−1.67	5.18*E* − 05
plasminogen activator, urokinase receptor; uPAR	Plaur	−1.59	3.80*E* − 04
Cyclin-dependent kinase inhibitor 2B (p15, inhibits CDK4); INK4b; p15	Cdkn2b	−1.47	4.66*E* − 03
Discoidin domain receptor family, member 2	Ddr2	−1.46	2.14*E* − 03
Angiomotin	Amot	−1.16	8.07*E* − 02
Thrombospondin 1	Thbs1	−1.13	1.48*E* − 01
Nuclear receptor subfamily 2, group F, member 2	Nr2f2	−1.10	2.62*E* − 01
Basic helix-loop-helix family, member e41; Sharp1; Bhlhb3; Bhlhb2l	Bhlhe41	−1.09	2.22*E* − 01
Smad family member 7	Smad7	−1.07	5.07*E* − 01
NME/NM23 nucleoside diphosphate kinase 1	Nme1	−1.03	6.95*E* − 01
Insulin-like growth factor binding protein 5	IGFBP5	−1.03	7.56*E* − 01
Integrin alpha 5 (fibronectin receptor alpha)	Itga5	−1.02	8.71*E* − 01
Mitogen-activated protein kinase kinase 7; MKK7	Map2k7	−1.01	9.48*E* − 01
Rho, GDP dissociation inhibitor (GDI) beta; RhoGD12	Arhgdib	−1.01	9.05*E* − 01
Mitogen-activated protein kinase 14; p38	Mapk14	−1.01	8.66*E* − 01
Eph receptor A5	EphA5	−1.01	9.06*E* − 01
Integrin beta 1 (fibronectin receptor beta)	Itgb1	1.01	9.52*E* − 01
Transformation related protein 53; p53	Trp53	1.02	7.07*E* − 01
Endothelial cell-specific molecule 1	Esm1	1.03	6.89*E* − 01
Mitogen-activated protein kinase kinase 4; MKK4	Map2k4	1.04	7.06*E* − 01
Tissue inhibitor of metalloproteinases 3	Timp3	1.04	6.82*E* − 01
Eph receptor B2; Erk	Ephb2	1.05	7.47*E* − 01
CD82 antigen; Kai1	Cd82	1.06	4.01*E* − 01
Mitogen-activated protein kinase kinase 6; MKK6	Map2k6	1.07	3.79*E* − 01
5′ Nucleotidase, ecto; CD73	Nt5e	1.08	4.20*E* − 01
Epidermal growth factor receptor	Egfr	1.11	3.53*E* − 01
Phosphatidylinositol 3-kinase, catalytic, beta polypeptide	PI3K (Pik3cb)	1.12	3.09*E* − 01
Twist basic helix-loop-helix transcription factor 2	Twist2	1.13	1.30*E* − 01
Mediator complex subunit 23; CRSP3	Med23	1.15	7.53*E* − 02
Lysophosphatidic acid receptor 1; Edg2	Lpar1	1.19	1.46*E* − 02
Breast cancer metastasis-suppressor 1-like	Brms1l	1.27	2.76*E* − 02
GATA binding protein 3	Gata3	1.34	1.45*E* − 02
Procollagen-proline, 2-oxoglutarate 4-dioxygenase (proline 4-hydroxylase), alpha 1 polypeptide	P4ha1	1.75	4.28*E* − 05

Genes with >2 times decrease or increase in expression and a *P* value < 0.05 were considered as significantly regulated. For Igf1r, Kiss1, and Hist1h2bk no data were available on the microarray.
